# The Diagnostic Value of the Vacuum Phenomenon during Hip Arthroscopy

**DOI:** 10.5402/2011/852390

**Published:** 2011-07-28

**Authors:** Ehud Rath, Yair Gortzak, Ran Schwarzkopf, Vadim Benkovich, Eugene Cohen, Dan Atar

**Affiliations:** ^1^Department of Orthopaedics, Soroka Medical Center, Ben-Gurion University of the Negev, Beer Sheva 84101, Israel; ^2^Department of Orthopaedic Surgery, Hospital for Joint Diseases, NYU Langone Medical Center 301 East 17th Street, New York, NY 10003, USA

## Abstract

The diagnostic value of the vacuum phenomenon between the femoral head and the acetabulum, and time frame of its occurrence after application of traction is an important clinical question. The resulting arthrogram may outline the shape, location, and extent of cartilage lesions prior to arthroscopy of the hip joint. The presence, duration, and diagnostic information of the vacuum phenomenon were evaluated in 24 hips that underwent arthroscopy. The operative diagnosis was compared to the results of imaging studies and to findings obtained during a traction trial prior to arthroscopy. Indications for arthroscopy included avascular necrosis, labral tears, loose bodies, osteoarthrosis, and intractable hip pain. In 22 hips the vacuum phenomenon developed within 30 seconds after application of traction. The most important data obtained from the vacuum phenomenon was the location and extent of femoral head articular cartilage detachment and the presence of nonossified loose bodies. The vacuum phenomenon did not reveal labral or acetabular cartilage pathology in any of these patients. The vacuum phenomenon obtained during the trial of traction can add valuable information prior to hip arthroscopy. Femoral head articular cartilage detachment was best documented by this method. The hip arthroscopist should utilize this diagnostic window routinely prior to hip arthroscopy.

## 1. Introduction

Hip arthroscopy, yet an infrequently performed procedure, has become more popular in recent years. The success of hip arthroscopy in treating hip pain depends on an accurate anatomical diagnosis. Multiple etiologies ranging between developmental, traumatic, and infectious can be responsible for hip pain. Arthroscopic inspection has identified many elusive sources of hip pathology. Consequently, there is an increasing awareness of various intraarticular lesions that can cause mechanical hip pain.

Early reports on arthroscopy of the hip centered on its ability to provide a diagnosis in occult, intra-articular pathology unrevealed on standard radiographic imaging such as computerized tomography and MRI [1, 2]. Yet as part of the procedure, before the surgical act and arthroscopy, a trial of traction is important in evaluating distractibility of the hip joint [3, 4]. A trial of traction opens a diagnostic window that may add valuable information for accurate diagnosis. Distraction of the hip creates a vacuum phenomenon between the femoral head and the acetabulum. A diastasis between the femoral head and the acetabulum is achieved by the application of traction on the lower extremity. The intra-articular low-pressure area can be visualized radiographically and is known as a “vacuum phenomenon.” The resulting arthrogram may outline the shape, location, and extent of cartilage lesions prior to arthroscopy of the hip joint. Inadequate distraction can result in traumatic entry of instruments into the hip joint and may result in cartilage scuffing or labrum avulsion. Traction is necessary for observing the innermost depths of the hip socket; without traction only a small portion of the intra-articular structures can be visualized [5]. 

The purpose of this paper was to document the incidence of the vacuum phenomenon, time for appearance, duration, and correlation with arthroscopic findings.

## 2. Materials and Methods

From August 2001 to January 2003, 24 hip arthroscopies were performed at our institution on 19 patients. The indications for arthroscopy included avascular necrosis of the femoral head, labral tears, loose bodies, osteoarthrosis, and intractable hip pain. Patient age ranged from 15 to 56 years. Diagnostic evaluation of each patient included plain radiographs of the pelvis and of the affected hip. Supplemental imaging studies such as magnetic resonance (MR) imaging and computed tomography (CT) scans were either obtained by the referring physician or performed at our institution. Physical examination, including range of motion and the presence of reproducible mechanical symptoms, was also reviewed. 

The procedure was performed under general anesthesia with the patient supine on a fracture table as described by Byrd [3]. Hip abduction of 25° aligned the traction force with the neck of the femur. The extremity was placed in neutral rotation. The contralateral extremity was abducted as necessary to accommodate the image intensifier being positioned between the legs.

Anterior-posterior, oblique, and axial fluoroscopic images were obtained before traction. Traction was limited to 25 kg to avoid any traction-related nerve injuries, pudendal nerve is the most common injured from excessive traction force [6]. Furthermore traction time was limited to less than two hours. Fluoroscopy was performed every thirty seconds from the beginning of traction until, appearance of the vacuum phenomenon. The presence and diagnostic information of the vacuum phenomenon was documented. 

The fluoroscopic findings were classified by their location in the hip joint as acetabular side, femoral side, or the intra-articular space.

All arthroscopies were performed using standard arthroscopic equipment. The hip was prepared and draped. The joint was distended with 20 mL of saline and the intracapsular position was confirmed by backflow through the needle. Access to the joint was obtained by using standard portals (anterior, anterolateral, and posterolateral) and controlled by fluoroscopy. The anterolateral portal was placed first for the introduction of the arthroscope. A 6-inch 14 G needle and a nitinol rod were inserted into the joint. A cannulated drill on a T handle was used to penetrate the firm hip capsule. The drill was slid into the joint on the nitinol rod by gentle oscillating motion to prevent iatrogenic chondral damage. As the drill penetrated the capsule the arthroscope sheath was inserted into the joint over the drill. The position of the scope was confirmed under fluoroscopy. Creation of the anterior portal was facilitated by direct visualization through the arthroscope as well as fluoroscopy. Once two portals were established instruments could be switched from one portal to the other by using a switching stick. This facilitated the systematic examination and the operative arthroscopy of the hip joint. A posterolateral portal was created when indicated.

The operative findings were compared to the imaging studies and to the findings obtained by the vacuum phenomenon during the trial of traction, which was conducted prior to the operation.

## 3. Results

In 22 hips the vacuum phenomenon developed within 30 seconds after application of traction. Two hips failed to distract—one with Otto—pelvis and one with osteoarthritis. These are the only cases in which the vacuum phenomenon was not evident. The vacuum phenomenon disappeared as the joint was inflated with saline. We did not measure the total time of presence of the vacuum phenomenon as prolonged traction could jeopardise the patients and predispose them to complications. The vacuum phenomenon increased within the first few minutes of each case as further distraction of the joint was accomplished. Distraction increased within the first few minutes without additional traction by overcoming the elastic properties of the capsule and ligament complex of the hip joint. 

**Figure 1:**
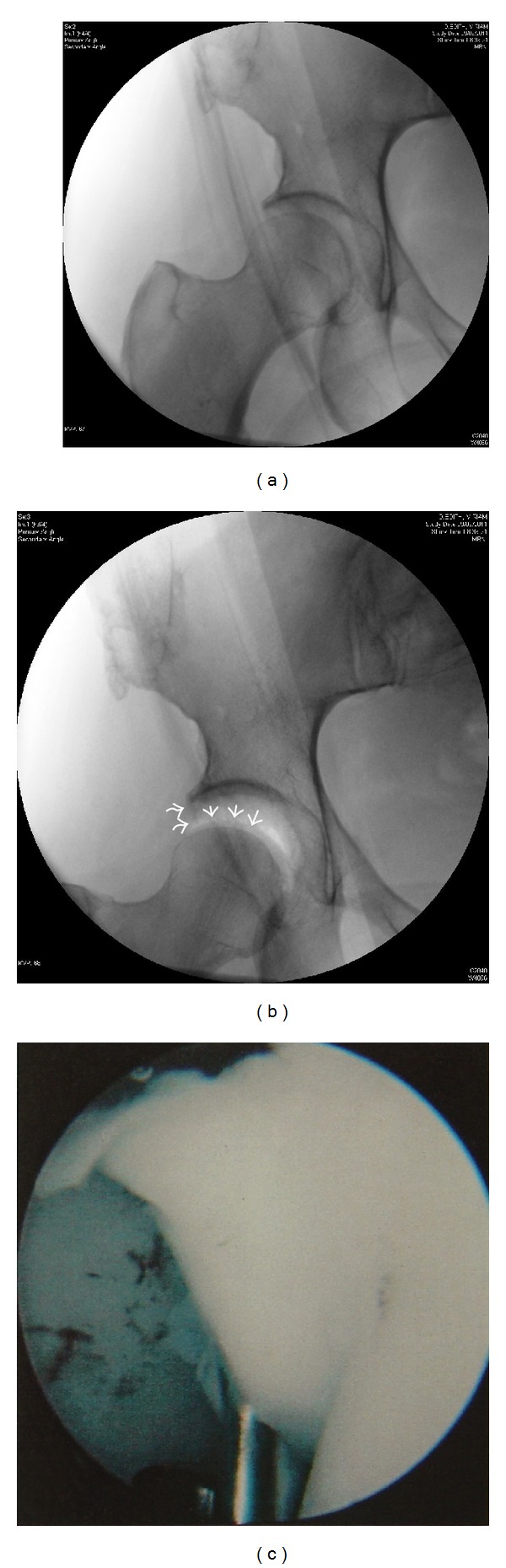
(a) Fluoroscopy of the right hip in 33-year-old male with a painful hip prior to traction. (b) One minute of traction: the vacuum phenomenon shows detachment of cartilage from the femoral head (adapted with permission from [7]). (c) Arthroscopic view of the right hip of this patient demonstrates cartilage detachment of the femoral head.

The most important data obtained from the vacuum phenomenon were the location and extent of femoral head articular cartilage detachment and the presence of other space occupying tissues within the intra-articular space, such as non ossified loose bodies and synovial proliferation. With the negative image mode, the vacuum phenomenon was much more pronounced. Dynamic detachment of loose cartilage on the femoral head was better demonstrated by this mode. A low-density area between the loose cartilage and the femoral head confirmed cartilage detachment that was not demonstrated by other methods (Figures 1(a) and 1(b)) [7]. The extent of cartilage damage visualized during arthroscopy correlated with the preoperative vacuum phenomenon assessment (Figure 1(c)). 

The vacuum phenomenon did not allow discovering labral or acetabular cartilage pathology in any of these patients. The extent of cartilage damage in these patients was not appreciated preoperatively by using standard imaging techniques (i.e., radiographs, CT, and MRI).

In 40% (10/22) of the patients there were major findings on arthroscopy that were not apparent prior to surgery—five labral tears, two cases of chondrolysis, one acetabular cyst, one ruptured ligamentum teres, and one massive cartilage detachment.

**Figure 2:**
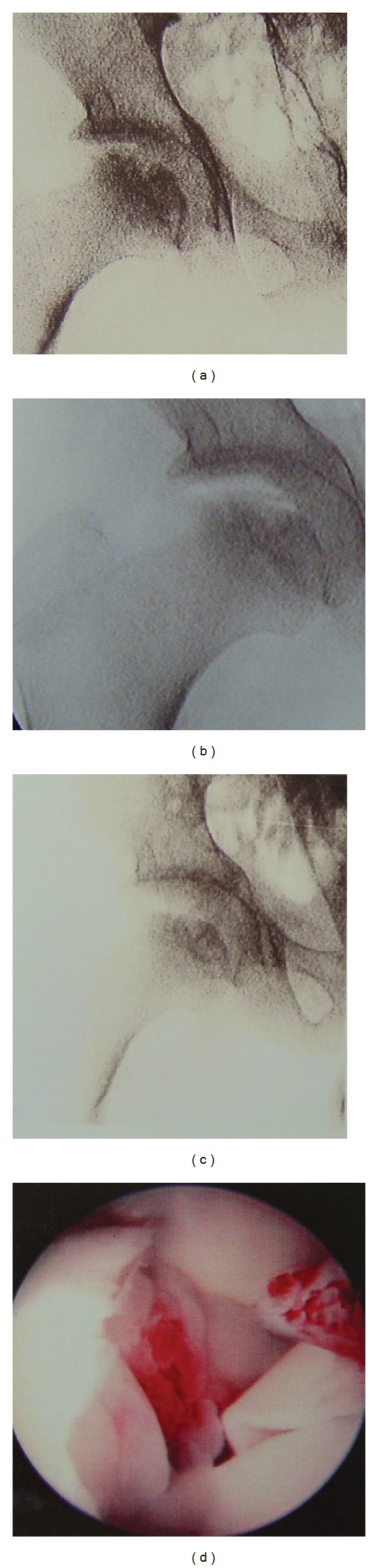
(a) Fluoroscopy of the right hip of a 15-year-old boy with Perthes disease prior to traction. (b) One minute of traction: the vacuum phenomenon shows a “double bubble” effect—a dynamic detachment of cartilage from the femoral head that was not evident on MRI. Vacuum fails to develop in the medial half of the joint. (c) Eight minutes of traction: the hip was further distracted and the vacuum phenomenon disappeared. (d) Arthroscopic view of the right hip shows chondrolysis of the femoral head. Massive synovial proliferation obliterates the medial aspect of the joint.

Synovial proliferation was also well demonstrated with the vacuum phenomenon. In one case of Legg-Calve-Perthes' disease the vacuum phenomenon demonstrated the presence of synovial proliferation in both the acetabular fossa and the inner wall of the capsule as well as osteochondritis dissecans of the femoral head (Figures 2(a)–2(d)).

One patient developed pudendal palsy, which resolved spontaneously within 3 months from surgery. No infections or other major complications have been described.

## 4. Discussion

Little has been written about the vacuum phenomenon in the hip joint. Sporadic papers related to the subject exist in the medical literature, especially concerning pathological conditions in the pediatric hip [8–10].

The vacuum phenomenon has been described in several pathological and nonpathological situations over the past eighty years. The development of a gas collection within the vertebral disk occurs with advanced disk degeneration [11] degenerative abnormalities of the atlantoodontoid joint [12] and in association with vertebral fractures [13].

The vacuum phenomenon has been used as a diagnostic tool in the radiological evaluation of knee anatomy in the 1950s [14]. In the 1970s it has been first described in association with hip joint pathology. Pozanski and Martel succeeded in creating a vacuum phenomenon with hip joint traction in 45/51 healthy volunteers, while in seven hip joints with effusion no vacuum phenomenon appeared with the application of traction. They concluded that in the presence of joint effusion the intraarticular fluid equalises the negative pressure produced by traction and abolishes the appearance of the vacuum phenomenon [15]. 

In the early 1980s Vegter and Van Der Broek described the appearance of a vacuum phenomenon in the hip joint during normal radiographs and described its usefulness in delineating the thickness of the articular cartilage [16]. The appearance of a vacuum phenomenon has been thought to exclude the presence of fluid within the joint and as such has been used as an adjunct in the radiographic evaluation of septic arthritis of the hip joint [10, 15–17]. More recently the appearance of a spontaneous vacuum phenomenon in the knee has been correlated with MRI imaging of degenerative tears of the meniscus or cartilage abnormalities [18, 19].

With the popularisation of hip arthroscopy in recent years the vacuum phenomenon has been attributed to the negative intracapsular pressure caused by distraction. As the joint is distended with fluid during the time of surgery, the phenomenon disappears [3].

Most complications occurring from hip arthroscopy are related to traction. It is important to understand the inherent laxity index of each patient to modify distraction forces during surgery. Neuropraxias may result from too much force for a prolonged period of time [6, 20].

It is clear that the use of traction should receive the same consideration as the use of a tourniquet. Since we do not have a tensiometer to control the amount of traction, we keep surgery time to less than 1 hour. 

The above report is to our knowledge the first to focus on the diagnostic value of the vacuum phenomenon observed during the preoperative trial of traction and its correlation with intraoperative findings. We use the diagnostic window of this phenomenon as part of the imaging arsenal prior to surgery. It helps us to reach a definite diagnosis and determine the location and extent of the intra-articular lesion. Best clinical correlation was found with femoral head articular detachment, which was not appreciated during standard imaging techniques and arthrograms. Arthro-MRI is not readily available in the Israeli hospital setting thus was not used with our patient population, and in order to obtain a proper arthro-MRI exam and not limit oneself to labral pathology traction needs to be obtained during the exam. The vacuum phenomenon did not add information in patients with labral tears and chondral lesions of the acetabulum; however, it did further correlate with synovitis and nonossified loose bodies found on arthroscopy.

In conclusion, the vacuum phenomenon which appears during the application of traction on the hip joint during hip arthroscopy seems to be an additional diagnostic tool in cases where hip arthroscopy is performed and when standard imaging techniques fail to elucidate the diagnosis.
